# Threshold effects of the relationship between physical exercise and cognitive function in the short-sleep elder population

**DOI:** 10.3389/fnagi.2023.1214748

**Published:** 2023-06-22

**Authors:** Yanwei You, Yuquan Chen, Xiangyu Chen, Mengxian Wei, Jiahui Yin, Qi Zhang, Qiang Cao

**Affiliations:** ^1^Division of Sports Science and Physical Education, Tsinghua University, Beijing, China; ^2^School of Social Sciences, Tsinghua University, Beijing, China; ^3^Institute of Medical Information/Medical Library, Chinese Academy of Medical Sciences and Peking Union Medical College, Beijing, China; ^4^College of Traditional Chinese Medicine, Shandong University of Traditional Chinese Medicine, Jinan, China; ^5^Undergraduate Department, Taishan University, Tai’an, China; ^6^Department of Earth Sciences, Kunming University of Science and Technology, Kunming, China; ^7^School of Pharmacy, Macau University of Science and Technology, Taipa, Macao SAR, China

**Keywords:** cognitive function, physical exercise, short-sleep, elder population, NHANES, threshold effect

## Abstract

**Background:**

It has been demonstrated that elderly people’s cognitive capacities can be improved with exercise, and short sleep is linked to cognitive decline. However, the impact of physical exercise on cognitive performance in seniors who do not get enough sleep is largely unknown. This makes it an intriguing subject to explore further.

**Methods:**

This study consisted of elders (over 60 years old) who participated throughout the National Health and Nutrition Examination Survey’s 2011–2014 cycle (NHANES). Weighted linear regression model and restricted cubic splines analysis were performed to evaluate the association between physical exercise and cognitive function. In the end, 1,615 samples were scrutinized and the total number of weighted respondents was 28,607,569.

**Results:**

Results showed that in the Animal Fluency test and the Digit Symbol Substitution test, a positive association was found between physical exercise volume and scores in the fully adjusted model. A two-piecewise linear regression model was then applied to explore the threshold effect of exercise on cognitive performance. Before 960 and 800 MET-minutes/week, there were consistent positive relationship between exercise and scores of the Animal Fluency test [ß (95% CI): 0.233 (0.154, 0.312), *p* < 0.001] and Digit Symbol Substitution test [β (95% CI): 0.555 (0.332, 0.778), *p* < 0.001], respectively. However, there was a saturation effect where physical exercise volume reached the two inflection points.

**Conclusion:**

According to our research, the benefit of exercise did not always expand with the exercise volume increment under the short-sleep condition, which challenged existing knowledge. The short-sleep elder group could maintain cognitive performance with no more than 800 MET-minutes/week of physical exercise. Verification of these findings requires further biological investigations.

## Graphical Abstract

Study design and main findings. 
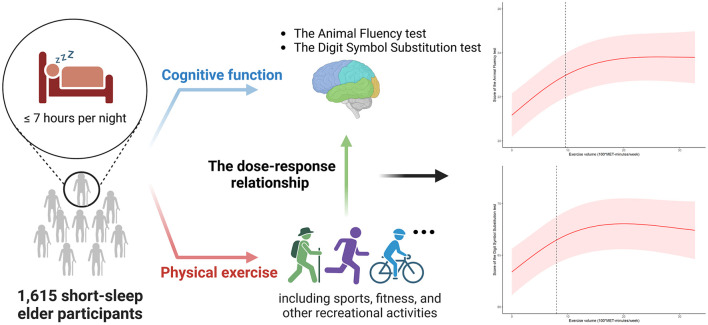

## 1. Introduction

Aging leads to cognitive decline such as progressive impairment in memory, judgment, language, and attention, among other cognitive domains (Morley, 2018). In United States, there was an increase in the prevalence of cognitive impairment among women from 18.7% in 1996 to 21.2% in 2014 and among men from 17.6% in 1996 to 21.0% in 2014 (Hale et al., 2020). It was determined from results analyzing 160 studies in a meta-analysis that dementia occurred at a pooled incidence of 17.2 per 1,000 person in a year in elder population aged 60 years or older (Fiest et al., 2016). A decline in cognitive abilities, especially cognitive impairments and their associated diseases, can have a profound effect on an individual, his or her family, and society in general (Wubker et al., 2015; Connors et al., 2019). Sleep factors, with unusual sleep patterns (i.e., short-sleep), poor sleep quality, and sleep disorder (e.g., insomnia) were associated with occurrence of cognitive impairment in the elderly.

In modern society, short-sleep in the elder population has become increasingly common. Sleeplessness and disturbed sleep appear to increase as people age, along with a decrease in good quality nocturnal sleep (Ohayon et al., 2004; Basner et al., 2007). In addition, plentiful evidence has shown that inadequate sleep had negative consequences on cognitive function (Hu et al., 2017; Xu et al., 2021; You et al., 2023c). One cross-sectional study using the UK Biobank data reported that short-sleep (<7 h) was associated with a significant decline in cognitive abilities in the elderly (Kyle et al., 2017). Over a 3-year follow-up, another population-based analysis of adults over 50 found that individuals who complained for sleep issues suffered accelerated cognitive deterioration than those who didn’t (Jelicic et al., 2002). Given the significant breadth and impact of insufficient sleep on cognition among elders, there was an urgent need to find effective and practical solutions to these problems.

Studies have found that regular exercise and physical activity was an effective strategy to mitigate the cognitive decline in the elder group (Espeland et al., 2017; Dominguez et al., 2021). Physical exercise can improve memory, focus, and concentration (Chirles et al., 2017), as well as reduce the risk of neurological diseases (Rolland et al., 2008), cardiovascular diseases (Fletcher et al., 2005), diabetes (Sampath Kumar et al., 2019), and osteoarthritis (You et al., 2021b), which were all common among senior citizens. Furthermore, regular exercise has been demonstrated to improve overall mental health (Deslandes et al., 2009; You et al., 2021c), reduce stress (Stubbs et al., 2017), and improve sleep disturbance (You et al., 2023a), which can all have a positive effect on cognitive function. Hence, regular exercise is a simple and effective way to maintain cognitive health, decrease the prevalence of certain diseases, and elevate living standards for elderly people.

However, the effects of physical exercise on cognition in the elderly were not consistent. An epidemiological study showed that neither global nor domain-specific cognitive function improved with moderate-intensity physical exercise programs after 24 months (Sink et al., 2015). Contrarily, a single exercise session had no impact on cognition and even raised perceptions of stress (Hopkins et al., 2012). It is possible that these inconsistent findings are due to different study designs and confounding factors, particularly when the impact of sleep on elderly people is considered. Elderly people’s cognitive abilities can be improved through exercise, while short sleep is associated with cognitive decline. To sum up, it is not only crucial but also interesting to explore the relationship between physical exercise and cognitive function under short-sleep conditions. Additionally, the evidence from large population-based studies is limited.

To the best of our knowledge, there was limited prior evidence that specifically examined whether physical exercise affected cognitive function in the community of elder groups with short-sleep conditions. In this study, by using a general sample from the National Health and Nutrition Examination Survey (NHANES), we aimed to: (1) examine the relationship between physical exercise and cognitive function in the short-sleep elder population; and (2) quantify its dose-response form and further assess the relationship by threshold analysis.

## 2. Materials and methods

### 2.1. Design and participants

Study data were from the National Health and Nutrition Examination Survey (NHANES), a comprehensive population-based survey intended to gather information about civilians in the United States (US). A multistage probability sampling design was applied to derive a typical selection of non-institutionalized households through the NHANES, which has been collected on approximately 10,000 people every two years since 1999. A research procedure of NHANES was approved by the Institutional Review Board (IRB) of the National Center for Health Statistics (NCHS), with written informed consent obtained.

**FIGURE 1:**
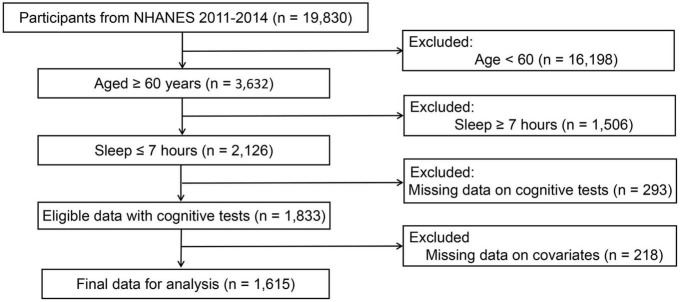
Flowchart depicting the selection strategy.

Based on two cycles of “continuous NHANES” (2011/2012, 2013/2014), a total of 3,632 participants were initially included after excluding those less than 60 years old (*n* = 16,198). In addition, participants who slept less than 7 h were included in this study, leaving 2,126 samples for finally analysis. Subsequently, eligible participants needed to have complete data on cognitive tests. This resulted in an analytical sample of 1,833 survey participants. Finally, participants without covariates data were excluded from the analysis, leaving 1,615 samples for finally analysis (see Figure 1).

### 2.2. Exposure measurement

Self-reported sleep length was collected on usual weekdays or workdays. In NHANES year cycle 2011–2014, participants were asked about their routine sleep hours: “How much sleep do you get (hours)?” Referring to previous literature (Su et al., 2021), short-sleep duration was defined as ≤7 h per night. The Physical Activity Questionnaire was used to gather data on the exposure variable, physical exercise, during home interviews. Physical exercise was defined as leisure time physical activity (including sports, fitness, and other recreational activities), as opposed to work-related physical activities (which included paid and unpaid jobs, household chores, and yard work).

The metabolic equivalent of task (MET) for the specified activity was multiplied by the participants’ reported weekly exercise time. To determine the MET-minutes per week, we used moderate and vigorous physical exercise (MVPE) measures. The Physical Activity Guidelines for Americans (PAGA) weighting mechanism was employed in the MVPE approach, where 2 min of moderate activity equated to 1 min of vigorous exercise (Ainsworth et al., 2000). Subsequently, the standard MET value of each activity was then multiplied by the overall amount of MVPE minutes per week to determine the MET-minutes per week. This method of quantifying physical exercise volume was also employed in earlier papers (You et al., 2022). Each level of exercise corresponded to a predetermined MET score, depending on whether reported as moderate (4 MET) or vigorous intensity (8 MET). Since the cumulative effects of a single exercise event may not be accurately reflected by a shift of 1 MET, exercise volume was described in terms of 100 MET as a unit of measurement (100*MET-min/week) in this study. In view of the fact that there was limited recommended physical activity guide for the elderly with short-sleep, the physical exercise volume was then categorized into three quantiles, none (<1 MET-min/week), low (from 1 to 360 MET-min/week), and moderate to vigorous (≥360 MET-min/week) for further analysis.

### 2.3. Cognitive function modules

The Animal Fluency test and the Digit Symbol Substitution test (DSST) were used to measure cognitive function in the NHANES. These assessments were conducted in a household interview or at a Mobile Examination Center (MEC). In the Animal Fluency test, which measured categorical verbal fluency and executive function, participants were instructed to name as many animals as they could in a minute. Processing speed, sustained attention, and working memory were evaluated using the Wechsler Adult Intelligence Scale (WAIS III) performance module called the DSST. This examination was administered using a paper form with a key located on top containing nine symbols and digits. A total of 133 symbols were displayed followed by numbers, and participants were asked to determine what each symbol represented in 2 min. Higher scores indicated greater cognitive function across all tests. The score was calculated as the total number of accurate matches. Detailed information about paradigm of the two cognitive tests was described in the Supplementary material.

### 2.4. Covariate assessment

Referring to the previous literature (Huang et al., 2021; You et al., 2023b), age, gender, race (non-Hispanic white, non-Hispanic black, Mexican American, and other races), marital status (never married, married or living with partner, widowed, divorced, or separated), family poverty income ratio [low income (<1), middle income (1,3), and high income (>3)], and educational attainment (below high school, high school, and college or above) were all extracted from the demographic questionnaire. Additionally, the questionnaires for smoking cigarettes and drinking alcohol were used to gather information about smoking status and alcohol intake status. According to the questionnaire replies, the status of alcohol use was divided into three categories: non-drinker, moderate alcohol use, and heavy alcohol use. Smoking status was classified as never, former, and current. Moreover, we evaluated the individuals’ chronic diseases (Wang et al., 2023). Participants were deemed to have diabetes mellitus (DM) if they met the following criteria: (1) A doctor has diagnosed you with diabetes; (2) HbA1c (%) > 6.5; (3) fasting glucose (mmol/l) ≥ 7.0; and (4) random blood glucose (mmol/l) ≥ 11.1; (5) 2-h OGTT Blood Glucose (mmol/l) ≥ 11.1; (6) use of insulin or diabetes drugs. Self-reported congestive heart failure, coronary heart disease, angina, heart attack, or stroke were attributed to cardiovascular disease (CVD). Detailed covariate information was available at http://www.cdc.gov/nchs/nhanes/.

### 2.5. Statistical analyses

To comply with the NHANES protocol, all data were combined into a single dataset and analyzed using the masked variance and weighting procedure. Using the weights from the Mobile Examination Center interviews, 4 years’ worth of survey data from NHANES 2011 to 2014 were combined to address non-response, non-coverage, and unequal probabilities of selection. This strategy was consistent with the weight method of prior researches (Shen et al., 2019; You et al., 2022). The merged weights were calculated as WT_11–14_ = (1/2) * WTMEC2YR_11–12_ + (1/2) * WTMEC2YR_13–14_, where WTMEC2YRs were variables from NHANES 2011–2014. In this study, we employed both multivariate adjusted and unadjusted models: Model 0 was adjusted for no covariates; Model 1 was adjusted for age, sex, race; Model 2 was adjusted for age, sex, race, marital status, education, poverty status, body mass index, smokers, alcohol drinkers, diabetes mellitus, and cardiovascular diseases.

Weighted linear regression model was used to investigate the association between physical exercise and test results for cognitive function. To explore the threshold impact and take into account any confounders, we constructed a model of two-piecewise linear regression. The threshold level of physical exercise (100 * MET-minutes/week) was determined using a recurrence method, which includes identifying the inflection point along a predefined interval and selecting the most likely inflection point. Using the log-likelihood ratio test, the two-piecewise linear regression model was compared to the one-line linear regression model. Simultaneously, the non-linear relationship was further evaluated using the restricted cubic spline (optimal knots = 3). Stratified analyses were conducted to explore the influence of covariates on the relationship between physical exercise and cognitive function. The R Foundation’s software^1^ was used for all statistical analyses, and a *p* value of 0.05 or less was regarded as statistically significant.

## 3. Results

In the final analysis, 1,615 participants aged 60 or older were included, and represented for a weighted population of 28,607,569. Table 1 displays the sociodemographic data of the study subjects. Study participants were an average of 68.79 years old, 47.17% of whom were male. The mean sleep length of all participants was 6.29 h per day. In addition, the average physical activity among total participants was 595 MET-minutes/week. For the cognitive function assessment, the mean score of the Animal Fluency test (reflecting verbal fluency and executive function) and the score of the Digit Symbol Substitution test (reflecting processing speed, sustained attention, and working memory) was 18.54 and 53.55, respectively.

**TABLE 1:** Demographic characteristics of study participants in NHANES.

Variable	(%/Mean)*
**Age**	
<65	36.42
[65, 72)	32.34
≥72	31.24
**Sex**	
Male	47.17
Female	52.83
**Race/ethnicity**	
Non-hispanic white	76.99
Non-hispanic black	9.32
Mexican American	3.68
Other race/ethnicity	10.01
**Marital status**	
Never married	4.63
Married/living with partner	64.90
Widowed/divorced	30.47
**Education**	
Below high school	5.65
High school	30.62
College or above	63.72
**Poverty income ratio**	
<1	9.47
[1,3)	37.11
≥3	53.42
**BMI (kg/m^2^)**	
<25	26.77
[25, 30)	34.41
≥30	38.82
**Smokers**	
Never smoker	50.96
Former smoker	37.24
Current smoker	11.80
**Alcohol drinkers**	
Non-drinker	36.11
Moderate alcohol use	57.68
High alcohol use	6.21
**Diabetes mellitus**	
No	68.81
Yes	31.19
**Cardiovascular diseases**	
No	79.42
Yes	20.58
Physical exercise (100*MET-minutes/week)	5.95 ± 0.48
Sleep duration (hours/day)	6.29 ± 0.03
Score of the Animal Fluency test	18.54 ± 0.26
Score of the Digit Symbol Substitution test	53.55 ± 0.67

*Weighted percentage for category variables and weighted Mean ± SE for continuous variables.

NHANES, National Health and Nutrition Examination Survey; BMI, body mass index; MET, metabolic equivalent of task.

Physical exercise and cognitive function test results were analyzed using a weighted linear regression model. As for the Animal Fluency test, when physical exercise was assessed as a continuous variable, Table 2 reveals that higher exercise volume was associated with better performance [Model 0, β (95% CI): 0.115 (0.085,0.144), *p* < 0.001; Model 1, β (95% CI): 0.099 (0.071, 0.128), *p* < 0.001; Model 2, β (95% CI): 0.077 (0.048, 0.106), *p* < 0.001]. Exercise was also associated with this outcome when assessed as a category variable. In the fully adjusted Model 2, taking the none exercise group as the reference, moderate to vigorous volume was positively associated with the Animal Fluency test scores [β (95% CI): 1.946 (1.126, 2.765), *p* < 0.001]. However, no significant association was identified in the low level physical exercise group. According to Supplementary Table 1, stratified analysis revealed that these associations were consistent across subgroups.

**TABLE 2:** Associations between physical exercise and cognitive function in the short-sleep elder population.

	Model 0^a^		Model 1^b^		Model 2^c^	
	**β (95% CI)**	***p*-value**	**β (95% CI)**	***p*-value**	**β (95% CI)**	***p*-value**
**Score of the Animal Fluency test**						
Physical exercise (100*MET-minutes/week)	0.115 (0.085,0.144)	<0.001	0.099 (0.071, 0.128)	<0.001	0.077 (0.048, 0.106)	<0.001
**Physical exercise (as category)**						
None	Reference		Reference		Reference	
Low	0.503 (−1.247,2.253)	0.561	0.294 (−1.227, 1.815)	0.694	0.160 (−1.308, 1.629)	0.813
Moderate to vigorous	2.746 (1.847,3.645)	<0.001	2.445 (1.596, 3.295)	<0.001	1.946 (1.126, 2.765)	<0.001
**Score of the Digit Symbol Substitution test**						
Physical exercise (100*MET-minutes/week)	0.263 (0.152, 0.375)	<0.001	0.224 (0.119, 0.328)	<0.001	0.077 (0.048, 0.106)	0.038
**Physical exercise (as category)**						
None	Reference		Reference		Reference	
Low	3.478 (−1.368, 8.325)	0.153	3.191 (−0.880, 7.262)	0.119	1.249 (−2.238, 4.736)	0.443
Moderate to vigorous	7.979 (4.952,11.007)	<0.001	6.684 (4.244, 9.123)	<0.001	3.707 (1.325, 6.090)	0.006

CI, confidence interval.

^a^Model 0, no covariates were adjusted.

^b^Model 1, age, sex, race were adjusted.

^c^Model 2, age, sex, race, marital status, education, poverty status, body mass index, smokers, alcohol drinkers, diabetes mellitus, and cardiovascular diseases were adjusted.

When it comes to the Digit Symbol Substitution test, similar findings are also identified in Table 2. Results showed that higher exercise volume was associated with higher scores of the Digit Symbol Substitution test when exercise volume was assessed as a continuous variable [Model 0, β (95% CI): 0.263 (0.152, 0.375), *p* < 0.001; Model 1, β (95% CI): 0.224 (0.119, 0.328), *p* < 0.001; Model 2, β (95% CI): 0.077 (0.048, 0.106), *p* = 0.038]. Also, this association persisted when exercise was assessed as a category variable. In the fully adjusted Model 2, using the reference group of those with no exercise, moderate to vigorous volume was positively associated with the Digit Symbol Substitution test performance [β (95% CI): 3.707 (1.325, 6.090), *p* = 0.006] in the Model 2. Additionally, a stratified analysis showed that these associations were consistent for subgroups with different demographic characteristics, as detailed in Supplementary Table 2.

**FIGURE 2:**
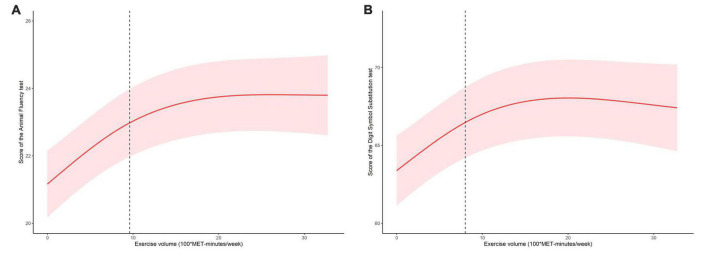
The dose-response relationship between exercise volume with the score of the Animal Fluency test **(A)** and score of the Digit Symbol Substitution test **(B)** in the short-sleep elders.

An analysis of the log-likelihood ratio was performed to compare the one-line (non-segmented) model to the segmented regression model, and our results indicated a threshold existed. As for the Animal Fluency test (Table 3), based on a two-piecewise linear regression model, we calculated that the inflection point was 960 MET-minutes/week. As seen on the left side of the inflection point, the β (95% CI) and *p*-value were 0.233 (0.154, 0.312) and < 0.001, respectively. On the right side of the inflection point, we found no significant association between physical exercise and cognitive test’s score, with β (95% CI) and *p*-value of 0.013 (−0.027, 0.053) and 0.522. Similar results are also found in the Digit Symbol Substitution test (Table 4). The score of the test was positively correlated with physical exercise until it bottomed out at 800 MET-minutes/week [β (95% CI): 0.555 (0.332, 0.778), *p* < 0.001]. However, when the physical exercise volume was higher than 800, such association seemed to saturate [β (95% CI): −0.044 (−0.136, 0.048), *p* = 0.349]. In Figure 2, using restricted cubic splines, we flexibly modeled and visualized the relationship between cognitive performance and physical exercise volume among short-sleep elders. When exercise volume reached 9.6 and 8 (100*MET-minutes/week), there was a saturation effect where the effects of physical exercise on the Animal Fluency test and Digit Symbol Substitution test plateaued.

**TABLE 3:** Threshold effect analysis of the relationship between physical exercise and score of the Animal Fluency test in the short-sleep elders (based on Model 2).

	β (95% CI)
One-line linear regression model	0.077 (0.051, 0.103)
Two-piecewise linear regression model	
Exercise < 9.6 (100*MET-minutes/week)	0.233 (0.154, 0.312)
Exercise ≥ 9.6 (100*MET-minutes/week)	0.013 (−0.027, 0.053)
Log-likelihood ratio test	

Age, sex, race, marital status, education, poverty status, body mass index, smokers, alcohol drinkers, diabetes mellitus, and cardiovascular diseases were adjusted.

**TABLE 4:** Threshold effect analysis of the relationship between physical exercise and score of the Digit Symbol Substitution test in the short-sleep elders (based on Model 2).

	β (95% CI)
One-line linear regression model	0.099 (0.037, 0.163)
Two-piecewise linear regression model	
Exercise < 8.0 (100*MET-minutes/week)	0.555 (0.332, 0.778)
Exercise ≥ 8.0 (100*MET-minutes/week)	−0.044 (−0.136, 0.048)
Log-likelihood ratio test	

Age, sex, race, marital status, education, poverty status, body mass index, smokers, alcohol drinkers, diabetes mellitus, and cardiovascular diseases were adjusted.

## 4. Discussion

In this population-based study, physical exercise volume was found to be positively associated with the cognitive function in the short-sleep elders. Additionally, exercise and cognitive performance were found to be non-linearly correlated. The relationship was stated as follows: the scores of cognitive tests rose substantially with the expanded level of exercise, but reached a plateau after exercise volume at 960 MET-minutes/week for the Animal Fluency test and 800 MET-minutes/week for the Digit Symbol Substitution test. Our study detected that physical exercise had a threshold effect on cognitive function in short-sleep elders.

Our study identified a positive association between physical exercise volume and cognitive function in elderly individuals who get short amounts of sleep, which was consistent with a prior meta-analytic study in the aging population (Colcombe and Kramer, 2003; Lam et al., 2018). In several population studies (Spirduso and Clifford, 1978; Emery and Gatz, 1990), it was also verified that older high-fit individuals performed cognitive tests better than older low-fit individuals. While the mechanism of this association was not yet understood, it was clear that, in addition to improving cognitive function, exercise has been linked to increased expression of brain chemicals such as molecular mediators and growth factors represented by brain-derived neurotrophic factor (BDNF). Animal studies showed that exercise induces BDNF in the brain, most robustly in the hippocampal region (Cotman et al., 2007). Neuronal cells’ survival, differentiation, migration, dendritic arborization, synaptogenesis, and plasticity were all influenced by BDNF (Greenberg et al., 2009). This neurotrophin’s molecular mechanism has been explored in a recent study that FNDC5, which was previously identified as a muscle protein induced by exercise, was elevated in the brain and might lead to better cognitive ability (Wrann et al., 2013). There was also a study that explored these effects from the molecular perspective that the benefits of exercise were attributed to the control of communications between BDNF, p-CREB, and NMDAR signaling, which was closely related to the brain function of spatial learning and memory (Wu et al., 2020).

From the dose-response investigation of the relation between exercise and cognitive function in short-sleep elders was performed, our study found that there was a threshold effect of exercise in this special group. The 2018 Physical Activity Guidelines indicated that there was still much to learn regarding how much physical exercise is necessary to enhance cognitive abilities (Erickson et al., 2019), and there lacked strong evidence that whether physical activity was always effective in improving the cognition, especially in short-sleep aged adults. Physical exercise appeared to have a threshold effect (no more than 800 MET-minutes/week) on cognitive function in short-sleep elders, with an increase in cognitive function observed when exercise volume was kept within such a level (800 MET-minutes/week volume exercise was equal to perform 200-min moderate intensity exercise or 100-minutes vigorous intensity exercise per week). This was a novel finding, considering that WHO Guidelines 2020 recommended elders to exercise for 150–300 min each week at a moderate level (WHO, 2020).

Consistent with our results, researchers using cohort data from the China Health and Retirement Longitudinal Study also discovered that moderate and mild physical exercise was linked to higher cognitive functioning, as opposed to vigorous exercise (Wu et al., 2021). Obviously, sleep plays an important role in cognitive function and lacking sleep may induce disorders of brain hormones and chemicals during daily executive activities. Moderate level exercise has been proposed to activate the reticulum system’s arousal mechanism, thus improving various cognitive functions (Dietrich and Audiffren, 2011). Excessive exercise might, however, result in the prefrontal cortex being disengaged from higher-order functions due to greater activation of the premotor cortex and supplementary motor area. It was also found that vigorous exercise might be difficult for beginners and elders with concomitant diseases, and could lead to feelings of incompetence, failure, and low self-esteem (You et al., 2021a). Hence, exercise should be regulated, taking into account the individual’s sleep habits and duration, to ensure the most beneficial effect on cognitive function.

The strengths of this study included that we used the study samples from a nationwide population. To our awareness, this was the initial research to examine the dose-response relationship between physical exercise and cognitive function in the specific short-sleep aged population. Exercise volume and cognitive performance associations were analyzed using adjusted weighted regression in consideration of the complex multistage sampling design of NHANES. In addition, threshold analysis was performed in order to quantify the dose-response form of the association. We also used the stratified analysis to further verify these results in consideration of confounding factors, including sociodemographic characteristics, BMI, smoking and alcohol drinking status, as well as chronic diseases.

It was also important to note that this study had the following limitations. Firstly, due to the NHANES’ cross-sectional design, the causal or temporal relationship among these associations was still questionable in the elderly short-sleep population. There was also an assumption that elders with cognitive decline or impairment would unable to perform high volume of exercise activities. Secondly, in spite of the fact that we adjusted for possible confounders, residual confounding effects (i.e., biological and genetic factors) could still bias our results. Thirdly, the measurement of physical exercise was assessed by self-report questionnaires in NHANES design, which tended to be imprecise compared with objectively measured test such as the accelerometer (Barros et al., 2021). Moreover, the results of this study were only applicable to elderly people with short sleep, and additional research is needed in order to better understand sleep patterns and the effects of age. Lastly, it is unknown whether the pandemic or other public health emergencies will alter these associations. Research on the biological mechanisms of exercise and COVID-19 is therefore necessary to shed light on this population’s cognition (Gonzales et al., 2022).

## 5. Conclusion

Utilizing the NHANES data, we assessed short-sleep older adults’ physical exercise and cognitive function in this study. Physical exercise showed positive associations with performance on a test of Animal Fluency and a test of Digit Symbol Substitution. In addition, a dose-response-based analysis detected the threshold effect in the short-sleep elders, and performing no more than 800 MET-minutes/week exercise was positively associated with cognitive abilities. However, the underlying molecular mechanisms of such association remain unknown. Future research is needed to better understand the relationship between physical exercise and cognitive function in this population, as well as the potential mechanisms.

## Data availability statement

Publicly available datasets were analyzed in this study. This data can be found here: https://wwwn.cdc.gov/nchs/nhanes/Default.aspx.

## Author contributions

YY, YC, and XC designed the study and wrote the original draft manuscript. YY, YC, XC, MW, and JY reviewed and edited the revised version of manuscript, collected, analyzed, and interpreted the data. QZ and QC critically administrated, reviewed, and approved the manuscript. All authors read and approved the final manuscript.

## Footnotes

1
http://www.R-project.org

## References

[B1] Ainsworth B. Haskell W. Whitt M. Irwin M. Swartz A. Strath S. (2000). Compendium of physical activities: An update of activity codes and met intensities. *Med. Sci. Sports Exerc.* 32 S498–S504. 10.1097/00005768-200009001-00009 10993420

[B2] Barros D. Borges-Machado F. Andrade da Silva W. Nascimento A. Carvalho J. Bohn L. (2021). Different subjective and objective measures and cut-points of physical activity in frailty phenotype screening: A need for standardization? *Arch. Gerontol. Geriatr.* 96:104479. 10.1016/j.archger.2021.104479 34274874

[B3] Basner M. Fomberstein K. Razavi F. Banks S. William J. Rosa R. (2007). American time use survey: Sleep time and its relationship to waking activities. *Sleep* 30 1085–1095. 10.1093/sleep/30.9.1085 17910380 PMC1978395

[B4] Chirles T. Reiter K. Weiss L. Alfini A. Nielson K. Smith J. (2017). Exercise training and functional connectivity changes in mild cognitive impairment and healthy elders. *J. Alzheimers Dis.* 57 845–856. 10.3233/JAD-161151 28304298 PMC6472271

[B5] Colcombe S. Kramer A. (2003). Fitness effects on the cognitive function of older adults: A meta-analytic study. *Psychol. Sci.* 14 125–130. 10.1111/1467-9280.t01-1-01430 12661673

[B6] Connors M. Seeher K. Teixeira-Pinto A. Woodward M. Ames D. Brodaty H. (2019). Mild cognitive impairment and caregiver burden: A 3-year-longitudinal study. *Am. J. Geriatr. Psychiatry* 27 1206–1215. 10.1016/j.jagp.2019.05.012 31230914

[B7] Cotman C. Berchtold N. Christie L. (2007). Exercise builds brain health: Key roles of growth factor cascades and inflammation. *Trends Neurosci.* 30 464–472. 10.1016/j.tins.2007.06.011 17765329

[B8] Deslandes A. Moraes H. Ferreira C. Veiga H. Silveira H. Mouta R. (2009). Exercise and mental health: Many reasons to move. *Neuropsychobiology* 59 191–198. 10.1159/000223730 19521110

[B9] Dietrich A. Audiffren M. (2011). The reticular-activating hypofrontality (Rah) model of acute exercise. *Neurosci. Biobehav. Rev.* 35 1305–1325. 10.1016/j.neubiorev.2011.02.001 21315758

[B10] Dominguez L. Veronese N. Vernuccio L. Catanese G. Inzerillo F. Salemi G. (2021). Nutrition, physical activity, and other lifestyle factors in the prevention of cognitive decline and dementia. *Nutrients* 13:4080. 10.3390/nu13114080 34836334 PMC8624903

[B11] Emery C. Gatz M. (1990). Psychological and cognitive effects of an exercise program for community-residing older adults. *Gerontologist* 30 184–188. 10.1093/geront/30.2.184 2347498

[B12] Erickson K. Hillman C. Stillman C. Ballard R. Bloodgood B. Conroy D. (2019). Physical activity, cognition, and brain outcomes: A review of the 2018 physical activity guidelines. *Med. Sci. Sports Exerc.* 51 1242–1251. 10.1249/MSS.0000000000001936 31095081 PMC6527141

[B13] Espeland M. Lipska K. Miller M. Rushing J. Cohen R. Verghese J. (2017). Effects of physical activity intervention on physical and cognitive function in sedentary adults with and without diabetes. *J. Gerontol. A Biol. Sci. Med. Sci.* 72 861–866. 10.1093/gerona/glw179 27590629 PMC6075086

[B14] Fiest K. Jette N. Roberts J. Maxwell C. Smith E. Black S. (2016). The prevalence and incidence of dementia: A systematic review and meta-analysis. *Can. J. Neurol. Sci.* 43 S3–S50. 10.1017/cjn.2016.18 27307127

[B15] Fletcher B. Gulanick M. Braun L. (2005). Physical activity and exercise for elders with cardiovascular disease. *Medsurg. Nurs.* 14 101–109. 15916265

[B16] Gonzales A. Lin J. Cha J. (2022). Physical activity changes among office workers during the covid-19 pandemic lockdown and the agreement between objective and subjective physical activity metrics. *Appl. Ergon.* 105:103845. 10.1016/j.apergo.2022.103845 35930899 PMC9296707

[B17] Greenberg M. Xu B. Lu B. Hempstead B. (2009). New insights in the biology of bdnf synthesis and release: Implications in cns function. *J. Neurosci.* 29 12764–12767. 10.1523/JNEUROSCI.3566-09.2009 19828787 PMC3091387

[B18] Hale J. Schneider D. Gampe J. Mehta N. Myrskyla M. (2020). Trends in the risk of cognitive impairment in the United States, 1996-2014. *Epidemiology* 31 745–754. 10.1097/EDE.0000000000001219 32740472 PMC7386871

[B19] Hopkins M. Davis F. Vantieghem M. Whalen P. Bucci D. (2012). Differential effects of acute and regular physical exercise on cognition and affect. *Neuroscience* 215 59–68. 10.1016/j.neuroscience.2012.04.056 22554780 PMC3374855

[B20] Hu M. Zhang P. Li C. Tan Y. Li G. Xu D. (2017). Sleep disturbance in mild cognitive impairment: A systematic review of objective measures. *Neurol. Sci.* 38 1363–1371. 10.1007/s10072-017-2975-9 28455768

[B21] Huang Y. Xu P. Fu X. Ren Z. Cheng J. Lin Z. (2021). The effect of triglycerides in the associations between physical activity, sedentary behavior and depression: An interaction and mediation analysis. *J. Affect. Disord.* 295 1377–1385. 10.1016/j.jad.2021.09.005 34565593

[B22] Jelicic M. Bosma H. Ponds R. Van Boxtel M. Houx P. Jolles J. (2002). Subjective sleep problems in later life as predictors of cognitive decline. Report from the Maastricht ageing study (MAAS). *Int. J. Geriatr. Psychiatry* 17 73–77. 10.1002/gps.529 11802234

[B23] Kyle S. Sexton C. Feige B. Luik A. Lane J. Saxena R. (2017). Sleep and cognitive performance: Cross-sectional associations in the UK biobank. *Sleep Med.* 38 85–91. 10.1016/j.sleep.2017.07.001 29031762 PMC5930168

[B24] Lam F. Huang M. Liao L. Chung R. Kwok T. Pang M. (2018). Physical exercise improves strength, balance, mobility, and endurance in people with cognitive impairment and dementia: A systematic review. *J. Physiother.* 64 4–15. 10.1016/j.jphys.2017.12.001 29289581

[B25] Morley J. (2018). An overview of cognitive impairment. *Clin. Geriatr. Med.* 34 505–513. 10.1016/j.cger.2018.06.003 30336985

[B26] Ohayon M. Carskadon M. Guilleminault C. Vitiello M. (2004). Meta-analysis of quantitative sleep parameters from childhood to old age in healthy individuals: Developing normative sleep values across the human lifespan. *Sleep* 27 1255–1273. 10.1093/sleep/27.7.1255 15586779

[B27] Rolland Y. Abellan van Kan G. Vellas B. (2008). Physical activity and Alzheimer’s disease: From prevention to therapeutic perspectives. *J. Am. Med. Dir. Assoc.* 9 390–405. 10.1016/j.jamda.2008.02.007 18585641

[B28] Sampath Kumar A. Maiya A. Shastry B. Vaishali K. Ravishankar N. Hazari A. (2019). Exercise and insulin resistance in type 2 diabetes mellitus: A systematic review and meta-analysis. *Ann. Phys. Rehabil. Med.* 62 98–103. 10.1016/j.rehab.2018.11.001 30553010

[B29] Shen L. Huang C. Lu X. Xu X. Jiang Z. Zhu C. (2019). Lower dietary fibre intake, but not total water consumption, is associated with constipation: A population-based analysis. *J. Hum. Nutr. Diet.* 32 422–431. 10.1111/jhn.12589 31087475

[B30] Sink K. Espeland M. Castro C. Church T. Cohen R. Dodson J. (2015). Effect of a 24-month physical activity intervention vs health education on cognitive outcomes in sedentary older adults: The life randomized trial. *JAMA* 314 781–790. 10.1001/jama.2015.9617 26305648 PMC4698980

[B31] Spirduso W. Clifford P. (1978). Replication of age and physical activity effects on reaction and movement time. *J. Gerontol.* 33 26–30. 10.1093/geronj/33.1.26 618962

[B32] Stubbs B. Vancampfort D. Rosenbaum S. Firth J. Cosco T. Veronese N. (2017). An examination of the anxiolytic effects of exercise for people with anxiety and stress-related disorders: A meta-analysis. *Psychiatry Res.* 249 102–108. 10.1016/j.psychres.2016.12.020 28088704

[B33] Su Y. Li C. Long Y. He L. Ding N. (2021). Association between bedtime at night and systolic blood pressure in adults in Nhanes. *Front. Med.* 8:734791. 10.3389/fmed.2021.734791 35004716 PMC8738078

[B34] Wang Y. Shen R. Ge J. (2023). Association between self-reported snoring and metabolic-associated fatty liver disease: A cross-sectional analysis of the Nhanes 2017-2018. *Sleep Med.* 101 414–420. 10.1016/j.sleep.2022.11.029 36516525

[B35] WHO (2020). *WHO Guidelines on physical activity and sedentary behaviour.* Geneva: Who Guidelines Approved by the Guidelines Review Committee.

[B36] Wrann C. White J. Salogiannnis J. Laznik-Bogoslavski D. Wu J. Ma D. (2013). Exercise induces hippocampal BDNF through a PGC-1ALPHA/FNDC5 pathway. *Cell Metab.* 18 649–659. 10.1016/j.cmet.2013.09.008 24120943 PMC3980968

[B37] Wu Y. Deng F. Wang J. Liu Y. Zhou W. Qu L. (2020). Intensity-dependent effects of consecutive treadmill exercise on spatial learning and memory through the P-CREB/BDNF/NMDAR signaling in hippocampus. *Behav. Brain Res.* 386:112599. 10.1016/j.bbr.2020.112599 32184158

[B38] Wu Z. Zhang H. Miao X. Li H. Pan H. Zhou D. (2021). High-intensity physical activity is not associated with better cognition in the elder: Evidence from the china health and retirement longitudinal study. *Alzheimers Res. Ther.* 13:182. 10.1186/s13195-021-00923-3 34732248 PMC8567563

[B39] Wubker A. Zwakhalen S. Challis D. Suhonen R. Karlsson S. Zabalegui A. (2015). Costs of care for people with dementia just before and after nursing home placement: Primary data from eight European countries. *Eur. J. Health Econ.* 16 689–707. 10.1007/s10198-014-0620-6 25069577

[B40] Xu W. Bai A. Huang X. Gao Y. Liu L. (2021). Association between sleep and motoric cognitive risk syndrome among community-dwelling older adults: Results from the china health and retirement longitudinal study. *Front. Aging Neurosci.* 13:774167. 10.3389/fnagi.2021.774167 PMC8641045 34867301

[B41] You Y. Chen Y. Yin J. Zhang Z. Zhang K. Zhou J. (2022). Relationship between leisure-time physical activity and depressive symptoms under different levels of dietary inflammatory index. *Front. Nutr.* 9:983511. 10.3389/fnut.2022.983511 36159493 PMC9490084

[B42] You Y. Liu J. Tang M. Wang D. Ma X. (2021b). Effects of Tai Chi exercise on improving walking function and posture control in elderly patients with knee osteoarthritis: A systematic review and meta-analysis. *Medicine* 100:e25655. 10.1097/MD.0000000000025655 33879749 PMC8078456

[B43] You Y. Wang D. Wang Y. Li Z. Ma X. A. (2021c). Bird’s-Eye view of exercise intervention in treating depression among teenagers in the last 20 years: A bibliometric study and visualization analysis. *Front. Psychiatry* 12:661108. 10.3389/fpsyt.2021.661108 34220574 PMC8249759

[B44] You Y. Li W. Liu J. Li X. Fu Y. Ma X. (2021a). Bibliometric review to explore emerging high-intensity interval training in health promotion: A new century picture. *Front. Public Health* 9:697633. 10.3389/fpubh.2021.697633 34368063 PMC8342813

[B45] You Y. Liu J. Wang D. Fu Y. Liu R. Ma X. (2023c). Cognitive performance in short sleep young adults with different physical activity levels: A cross-sectional fNIRS study. *Brain Sci.* 13:171. 10.3390/brainsci13020171 36831714 PMC9954673

[B46] You Y. Chen Y. Fang W. Li X. Wang R. Liu J. (2023a). The association between sedentary behavior, exercise, and sleep disturbance: A mediation analysis of inflammatory biomarkers. *Front. Immunol.* 13:1080782. 10.3389/fimmu.2022.1080782 36713451 PMC9880546

[B47] You Y. Chen Y. Zhang Q. Yan N. Ning Y. Cao Q. (2023b). Muscle quality index is associated with trouble sleeping: A cross-sectional population based study. *BMC Public Health* 23:489. 10.1186/s12889-023-15411-6 36918831 PMC10012435

