# Supplementary material for: Threshold effects of the relationship between physical exercise and cognitive function in the short-sleep elder population
Source: Front Aging Neurosci. 2023 Jun 22;15:1214748. doi: 10.3389/fnagi.2023.1214748 (PMC10323428; doi:10.3389/fnagi.2023.1214748)
Supplement: Supplementary file 1 [file Data_Sheet_1.docx]

**Paradigm of the two cognitive test in NHANES 2011-2014 (as described in the NHANES CFQ questionnaire).**

The Animal Fluency test examines categorical verbal fluency, a component of executive function. The test demands awareness (e.g., naming animals), regardless of cultural context, that is not absolutely reliant on formal educational experiences of a particular culture. The Animal Fluency test has been used in large-scale screenings and epidemiologic studies. Participants are asked to name as many animals as possible in one minute. A point is given for each named animal. In NHANES, participants first were asked to name three items of clothing, another verbal fluency category, as a practice test. Participants who could not name three articles of clothing did not continue with the Animal Fluency exercise.

The Digit Symbol Substitution test (DSST), a performance module from the Wechsler Adult Intelligence Scale (WAIS III), relies on processing speed, sustained attention, and working memory. The DSST has been used in large screenings, epidemiological and clinical studies, and was administered during the household interview to participants 60 years and over during NHANES 1999-2002. The exercise is conducted using a paper form that has a key at the top containing 9 numbers paired with symbols. Participants have 2 minutes to copy the corresponding symbols in the 133 boxes that adjoin the numbers. The score is the total number of correct matches. A sample practice test is administered before the participants begin the main test. In NHANES, participants who could not correctly match the symbols with the numbers on their own during the pretest practice did not continue.

Table S1. Weighted linear regression of stratified results for physical exercise with the score of the animal fluency test.

|  | None exercise | Low volume exercise | p | Moderate to vigorous volume exercise | p | p for trend |
| --- | --- | --- | --- | --- | --- | --- |
| Sex |  |  |  |  |  |  |
| Male | Reference | 1.035(-1.114,3.184) | 0.333 | 2.73( 1.527,3.932) | <0.001 | <0.001 |
| Female | Reference | 0.866(-1.018,2.751) | 0.355 | 2.862( 1.562,4.162) | <0.001 | <0.001 |
| Age |  |  |  |  |  |  |
| < 65 | Reference | 1.151(-1.145,3.446) | 0.314 | 2.913( 1.366,4.460) | <0.001 | <0.001 |
| [65, 72) | Reference | 0.508(-1.759,2.774) | 0.650 | 1.883( 0.343,3.422) | 0.018 | 0.019 |
| ≥72 | Reference | 0.125(-2.676,2.927) | 0.928 | 2.635( 1.185,4.085) | <0.001 | <0.001 |
| Race/ethnicity |  |  |  |  |  |  |
| Non-hispanic White | Reference | 1.297(-0.624,3.219) | 0.178 | 3.149( 2.034,4.263) | <0.001 | <0.001 |
| Non-hispanic Black | Reference | 1.234(-0.377,2.845) | 0.127 | 2.421( 1.000,3.842) | 0.002 | 0.002 |
| Mexican American | Reference | 2.085(-1.502,5.671) | 0.212 | 1.353(-1.496,4.202) | 0.298 | 0.266 |
| Other race/ethnicity | Reference | -0.71(-3.288,1.868) | 0.576 | 0.971(-1.112,3.054) | 0.347 | 0.361 |
| Marital status |  |  |  |  |  |  |
| Never married | Reference | -1.146(-5.084,2.792) | 0.550 | 0.395(-3.934,4.724) | 0.851 | 0.856 |
| Married/living with partner | Reference | 0.495(-1.136,2.126) | 0.540 | 3.093( 2.259,3.926) | <0.001 | <0.001 |
| Widowed/ divorced | Reference | 2.088(-1.112,5.287) | 0.193 | 2.271( 0.566,3.976) | 0.011 | 0.010 |
| Poverty income ratio |  |  |  |  |  |  |
| <1 | Reference | 2.302(-1.182,5.785) | 0.186 | 0.617(-1.074,2.308) | 0.461 | 0.322 |
| [1,3) | Reference | -0.137(-2.065,1.792) | 0.886 | 2.109( 0.415,3.803) | 0.016 | 0.018 |
| ≥3 | Reference | 0.782(-1.233,2.797) | 0.434 | 2.821( 1.630,4.012) | <0.001 | <0.001 |
| Education |  |  |  |  |  |  |
| Below high school | Reference | 0.003(-1.373,1.379) | 0.997 | 0.067(-2.067,2.201) | 0.949 | 0.949 |
| High school | Reference | -0.757(-2.685,1.172) | 0.429 | 1.083( 0.142,2.025) | 0.026 | 0.050 |
| College or above | Reference | 1.293(-0.619,3.205) | 0.178 | 2.373( 1.228,3.518) | <0.001 | <0.001 |
| BMI (kg/m^2^) |  |  |  |  |  |  |
| <25 | Reference | -1.739(-4.283,0.805) | 0.173 | 3.304( 1.901,4.708) | <0.001 | <0.001 |
| [25, 30) | Reference | 1.857(-0.071,3.784) | 0.058 | 2.991( 1.727,4.255) | <0.001 | <0.001 |
| ≥30 | Reference | 1.033(-1.173,3.239) | 0.347 | 2.205( 0.365,4.046) | 0.020 | 0.016 |
| Smokers |  |  |  |  |  |  |
| Never smoker | Reference | 1.045(-0.924,3.015) | 0.287 | 2.923( 1.642,4.203) | <0.001 | <0.001 |
| Former smoker | Reference | 1.217(-0.966,3.400) | 0.264 | 2.644( 1.197,4.090) | <0.001 | <0.001 |
| Current smoker | Reference | -0.459(-5.880,4.963) | 0.864 | 2.275( 0.178,4.373) | 0.035 | 0.073 |
| Alcohol drinkers |  |  |  |  |  |  |
| Nondrinker | Reference | 1.014(-1.508,3.537) | 0.418 | 1.864( 0.526,3.202) | 0.008 | 0.008 |
| Moderate alcohol use | Reference | 0.424(-1.742,2.591) | 0.692 | 2.752( 1.450,4.053) | <0.001 | <0.001 |
| High alcohol use | Reference | 1.307(-6.064,8.678) | 0.716 | 3.006( 0.375,5.637) | 0.027 | 0.028 |
| Cardiovascular diseases |  |  |  |  |  |  |
| No | Reference | 1.23(-0.573,3.033) | 0.174 | 2.935( 1.833,4.037) | <0.001 | <0.001 |
| Yes | Reference | 0.159(-2.139,2.458) | 0.888 | 1.663(-0.067,3.392) | 0.059 | 0.063 |
| Diabetes mellitus |  |  |  |  |  |  |
| No | Reference | 1.72(-0.323,3.763) | 0.096 | 1.542(-0.225,3.309) | 0.085 | 0.053 |
| Yes | Reference | 1.007(-1.198,3.213) | 0.358 | 3.397( 2.441,4.352) | <0.001 | <0.001 |

Table S2. Weighted linear regression of stratified results for physical exercise with the score of the Digit Symbol Substitution test.

|  | None exercise | Low volume exercise | p | Moderate to vigorous volume exercise | p | p for trend |
| --- | --- | --- | --- | --- | --- | --- |
| Sex |  |  |  |  |  |  |
| Male | Reference | 5.09(1.036, 9.143) | 0.016 | 7.598(3.728,11.467) | <0.001 | <0.001 |
| Female | Reference | 4.069(-1.398, 9.536) | 0.139 | 8.539( 5.157,11.920) | <0.001 | <0.001 |
| Age |  |  |  |  |  |  |
| < 65 | Reference | 4.139(-0.699, 8.977) | 0.091 | 5.725( 0.581,10.869) | 0.030 | 0.029 |
| [65, 72) | Reference | 0.954(-3.402, 5.310) | 0.658 | 8.504( 4.949,12.059) | <0.001 | <0.001 |
| ≥72 | Reference | 4.71(-1.251,10.671) | 0.117 | 6.317( 3.047, 9.588) | <0.001 | <0.001 |
| Race/ethnicity |  |  |  |  |  |  |
| Non-hispanic White | Reference | 4.627(-0.304, 9.558) | 0.065 | 8.148( 4.667,11.630) | <0.001 | <0.001 |
| Non-hispanic Black | Reference | 7.251(2.145,12.358) | 0.007 | 5.974(0.716,11.233) | 0.028 | 0.02 |
| Mexican American | Reference | 12.285( 1.202,23.367) | 0.034 | 6.544(-0.403,13.491) | 0.061 | 0.046 |
| Other race/ethnicity | Reference | 3.563(-6.569,13.696) | 0.476 | 9.251( 2.624,15.878) | 0.008 | 0.008 |
| Marital status |  |  |  |  |  |  |
| Never married | Reference | -5.213(-20.151, 9.725) | 0.473 | 4.133( -7.570,15.836) | 0.468 | 0.476 |
| Married/living with partner | Reference | 5.973(2.089, 9.856) | 0.004 | 8.799(5.624,11.975) | <0.001 | <0.001 |
| Widowed/ divorced | Reference | 2.681(-4.269, 9.630) | 0.437 | 6.361( 1.273,11.449) | 0.016 | 0.017 |
| Poverty income ratio |  |  |  |  |  |  |
| <1 | Reference | 0.333(-7.661,8.326) | 0.932 | 5.05( 0.646,9.453) | 0.026 | 0.030 |
| [1,3) | Reference | 5.565(-1.233,12.364) | 0.105 | 7.352( 3.724,10.980) | <0.001 | <0.001 |
| ≥3 | Reference | 1.053(-4.323,6.428) | 0.692 | 5.518( 1.601,9.435) | 0.007 | 0.008 |
| Education |  |  |  |  |  |  |
| Below high school | Reference | 1.872(-3.392,7.136) | 0.471 | 1.789(-4.971,8.549) | 0.591 | 0.509 |
| High school | Reference | 1.059(-3.508,5.626) | 0.639 | 2.014(-1.807,5.834) | 0.290 | 0.257 |
| College or above | Reference | 4.289(-0.152,8.730) | 0.058 | 6.187( 2.576,9.798) | 0.001 | 0.001 |
| BMI (kg/m^2^) |  |  |  |  |  |  |
| <25 | Reference | -1.335(-8.942, 6.271) | 0.722 | 11.138( 5.375,16.901) | <0.001 | <0.001 |
| [25, 30) | Reference | 7.849(2.456,13.241) | 0.006 | 7.938(4.466,11.409) | <0.001 | <0.001 |
| ≥30 | Reference | 3.112(-2.086, 8.310) | 0.231 | 6.142( 1.969,10.315) | 0.005 | 0.004 |
| Smokers |  |  |  |  |  |  |
| Never smoker | Reference | 5.234(-0.906,11.373) | 0.092 | 8.259( 4.171,12.347) | <0.001 | <0.001 |
| Former smoker | Reference | 5.399(0.224,10.574) | 0.041 | 7.139(3.331,10.947) | <0.001 | <0.001 |
| Current smoker | Reference | -5.016(-14.104, 4.073) | 0.267 | 7.214( -0.035,14.463) | 0.051 | 0.128 |
| Alcohol drinkers |  |  |  |  |  |  |
| Nondrinker | Reference | 0.343(-6.076, 6.762) | 0.914 | 7.456( 2.983,11.930) | 0.002 | 0.002 |
| Moderate alcohol use | Reference | 3.772(-0.212, 7.755) | 0.063 | 6.274( 1.943,10.605) | 0.006 | 0.006 |
| High alcohol use | Reference | 11.462(-5.619,28.543) | 0.177 | 12.971( 0.466,25.476) | 0.043 | 0.039 |
| Cardiovascular diseases |  |  |  |  |  |  |
| No | Reference | 4.672(0.151, 9.193) | 0.043 | 6.981(3.114,10.848) | <0.001 | <0.001 |
| Yes | Reference | 4.125(-1.544, 9.794) | 0.147 | 9.236( 5.345,13.128) | <0.001 | <0.001 |
| Diabetes mellitus |  |  |  |  |  |  |
| No | Reference | 6.163(0.744,11.583) | 0.027 | 6.028(0.716,11.340) | 0.027 | 0.013 |
| Yes | Reference | 4.32(-1.994,10.635) | 0.173 | 7.934( 4.248,11.620) | <0.001 | <0.001 |
